# NFκB-Activated COX2/PGE_2_/EP4 Axis Controls the Magnitude and Selectivity of BCG-Induced Inflammation in Human Bladder Cancer Tissues

**DOI:** 10.3390/cancers13061323

**Published:** 2021-03-16

**Authors:** Omar M. Ibrahim, Per H. Basse, Weijian Jiang, Khurshid Guru, Gurkamal Chatta, Pawel Kalinski

**Affiliations:** 1 Department of Medicine and Center for Immunotherapy, Roswell Park Comprehensive Cancer Center, Buffalo, NY 14263, USA; oibrahim@buffalo.edu (O.M.I.); per.basse@roswellpark.org (P.H.B.); weijian.jiang@roswellpark.org (W.J.); 2 The Graduate School, University at Buffalo, The State University of New York, Buffalo, NY 14260, USA; 3 Department of Urology, Roswell Park Comprehensive Cancer Center, Buffalo, NY 14263, USA; khurshid.guru@roswellpark.org (K.G.); gurkamal.chatta@roswellpark.org (G.C.)

**Keywords:** chemokines, tumor microenvironment (TME), Bacillus Calmette–Guérin (BCG), bladder cancer, immunomodulation, immunotherapy, prostaglandin E_2_ (PGE_2_), effector T cells, regulatory T cells

## Abstract

**Simple Summary:**

The clinical effectiveness of Bacillus Calmette-Guérin (BCG) is limited to patients with early stages of bladder cancer (BlCa) and its effects are often transient. To understand the mechanisms limiting the effectiveness of BCG, we evaluated its impact on the human BlCa tumor microenvironment (TME) and the feasibility of its pharmacologic modulation. We observed that BCG non-selectively induces both CTL-attracting chemokines and Treg/MDSC attractants and suppressive factors in human BlCa tissue explants, in a mechanism involving NFκB-induced PGE_2_ synthesis and EP4 signaling. In contrast to non-selective impact of NFκB blockade on BCG-induced inflammation, the PGE_2_ antagonism selectively enhanced the BCG-driven production of CTL attractants but eliminated the induction of Treg/MDSC attractants and suppressive factors, enhancing the CTL migration but reducing Treg attraction to BCG-treated BlCa. Since intratumoral CTL accumulation predicts long term patient outcomes and the effectiveness of cancer immunotherapies, our data indicates the feasibility of targeting the PGE_2_-chemokine interplay to enhance the therapeutic effects of BCG.

**Abstract:**

Bacillus Calmette-Guérin (BCG) is commonly used in the immunotherapy of bladder cancer (BlCa) but its effectiveness is limited to only a fraction of patients. To identify the factors that regulate the response of human BlCa tumor microenvironment (TME) to BCG, we used the ex vivo whole-tissue explant model. The levels of COX2 in the BCG-activated explants closely correlated with the local production of Treg- and MDSCS attractants and suppressive factors, while the baseline COX2 levels did not have predictive value. Accordingly, we observed that BCG induced high levels of MDSC- and Treg-attracting chemokines (CCL22, CXCL8, CXCL12) and suppressive factors (IDO1, IL-10, NOS2). These undesirable effects were associated with the nuclear translocation of phosphorylated NFκB, induction of COX2, the key enzyme controlling PGE_2_ synthesis, and elevation of a PGE_2_ receptor, EP4. While NFκB blockade suppressed both the desirable and undesirable components of BCG-driven inflammation, the inhibitors of PGE_2_ synthesis (Celecoxib or Indomethacin) or signaling (EP4-selective blocker, ARY-007), selectively eliminated the induction of MDSC/Treg attractants and immunosuppressive factors but enhanced the production of CTL attractants, CCL5, CXCL9 and CXCL10. PGE_2_ blockade allowed for the selectively enhanced migration of CTLs to the BCG-treated BlCa samples and eliminated the enhanced migration of Tregs. Since the balance between the CTLs and suppressive cells in the TME predicts the outcomes in patients with BlCa and other diseases, our data help to elucidate the mechanisms which limit the effectiveness of BCG therapies and identify new targets to enhance their therapeutic effects.

## 1. Introduction

Bacillus Calmette–Guérin (BCG) is an attenuated live mycobacterium originally developed as a preventive tuberculosis vaccine [1,2]. Following the demonstration of its therapeutic activity in bladder cancer (BlCa) [3,4], BCG was approved by the FDA for the local treatment of non-muscle invasive BlCa (NMIBC) [5]. The intravesical administration of BCG stimulates pro-inflammatory cytokines, promoting the recruitment of immune cells [4,6,7,8,9]. Despite significant effectiveness of BCG in high-risk NMIBC patients, 31–78% of the initial BCG-responsive patients demonstrate disease recurrence and the development of BCG resistance [8,10,11]. The resulting muscle-invasive BlCa (MIBC) is treated with cystectomy and chemotherapy, which prolong survival, but significantly reduce the quality of life [11,12,13,14], highlighting the need for improved treatment modalities [9,15,16,17,18,19].

Tumor-infiltrating immune cells involve immunostimulatory and suppressive populations, with respective antitumor and tumor-promoting functions [20]. Infiltrating CD8^+^ cytotoxic T cells (CTLs) predict improved survival in bladder cancer and other tumors [21,22,23]. In addition, intratumoral CTLs are critically important for the clinical activity of immune checkpoint inhibition (ICI) blockers, such as PD-1, PD-L1, or CTLA-4 blocking antibodies [24,25,26]. In contrast, the preferential accumulation of regulatory T cells (Tregs) and myeloid-derived suppressor cells (MDSCs) predicts poor survival [27,28]. CTLs and suppressive cells are attracted to tumor tissues by different chemokines: Th1/NKs/CTLs-attracting chemokines include CCL5 (regulated upon activation, normal T cell expressed, and secreted; RANTES), CXCL9 (monokine induced by gamma interferon; MIG) and CXCL10 (Interferon gamma-induced protein 10; IP10), while neutrophils, MDSCs, and Tregs are attracted by, respectively, CXCL8 (interleukin 8; IL-8), CXCL12 (stromal-derived factor-1; SDF1) and CCL22 (myeloid-derived chemokine; MDC) [29,30,31,32]. Our own studies demonstrated that the balance between the production of CTL- versus Treg/MDSC attracting chemokines induced by poly-IC in colon and ovarian cancer tissues is affected by the key ubiquitous regulator and inflammatory mediator, PGE_2_ and the key enzyme controlling its production, COX2 [33,34]. This raises the possibility that the levels of PGE_2_ production and/or signaling at baseline or in response to BCG exposure may affect the balance between tumor-promoting and anti-tumor subsets of effector cells in the TME.

Guided by above considerations, correlations between high expression of COX2 in human BlCa tissues and tumor progression and treatment resistance [35,36,37,38,39,40,41], the ability of COX2 blocker, celecoxib, to counteract tumor growth in BlCa-bearing animals (either receiving BCG or not) [15,41,42,43] and our observations that CCL22, CXCL12, and CXCL8 are induced as an undesirable side effect of BCG in BlCa tissues [7], we evaluated the role of PGE_2_ and its mechanistic basis in the regulation of baseline and BCG-induced inflammation in human BlCa TME.

## 2. Results

### 2.1. BCG Induces Both the Effector-Type and Suppressive Chemokines and Inflammatory Mediators

**Figure 1:**
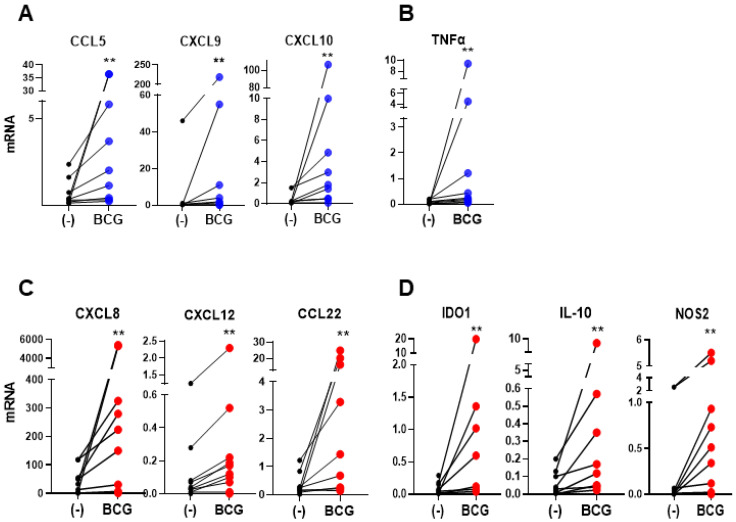
Bacillus Calmette-Guérin (BCG) induces CTL-attracting chemokines, but also MDSCs/Tregs-attracting chemokines and immunosuppressive inflammatory mediators in BlCa TME. The effect of BCG on the mRNA expression of ex vivo treated tumor tissues from BlCa patients (*n* = 9 different patients’ tissues; data are shown data relative to HPRT1). (**A**), BCG treatment enhances the expression of CTLs-attractants; CCL5, CXCL9, and CXCL10; and (**B**), pro-inflammatory cytokine TNFα, as well as (**C**), MDSCs/Tregs-attractants CXCL8, CXCL12, and CCL22, and (**D**), Immunosuppressive factors IDO1, IL-10 and NOS2. Each pair of symbols and the connecting line represents the data from a single patient, expressed as the mean mRNA expression levels of triplicate cultures per patient, relative to HPRT1. Wilcoxon rank test is used to determine statistical significance. ** *p* < 0.01.

In order to determine the ability of BCG to affect the chemokine production in the human bladder cancer microenvironment, post-surgical tumors from untreated bladder cancer patients that included all compartments of tumor environment were cultured ex vivo with or without BCG according to our established protocol [7,33]. Despite a significant heterogeneity of both baseline and inducible chemokine production, we observed that BCG uniformly elevated several CTL-attracting chemokines: CCL5, CXCL9, and CXCL10 (Figure 1A), but also strongly elevated the intratumoral expression of neutrophils-attracting chemokine CXCL8, MDSC- and Treg-attracting chemokine CXCL12, and Treg-attracting chemokine CCL22 (Figure 1C). In addition, BCG-treated tumors expressed high levels of the immunostimulatory factor TNFα (Figure 1B), but also of multiple immunosuppressive factors such as IDO1, IL-10 and NOS2 (Figure 1D).

**Figure 2:**
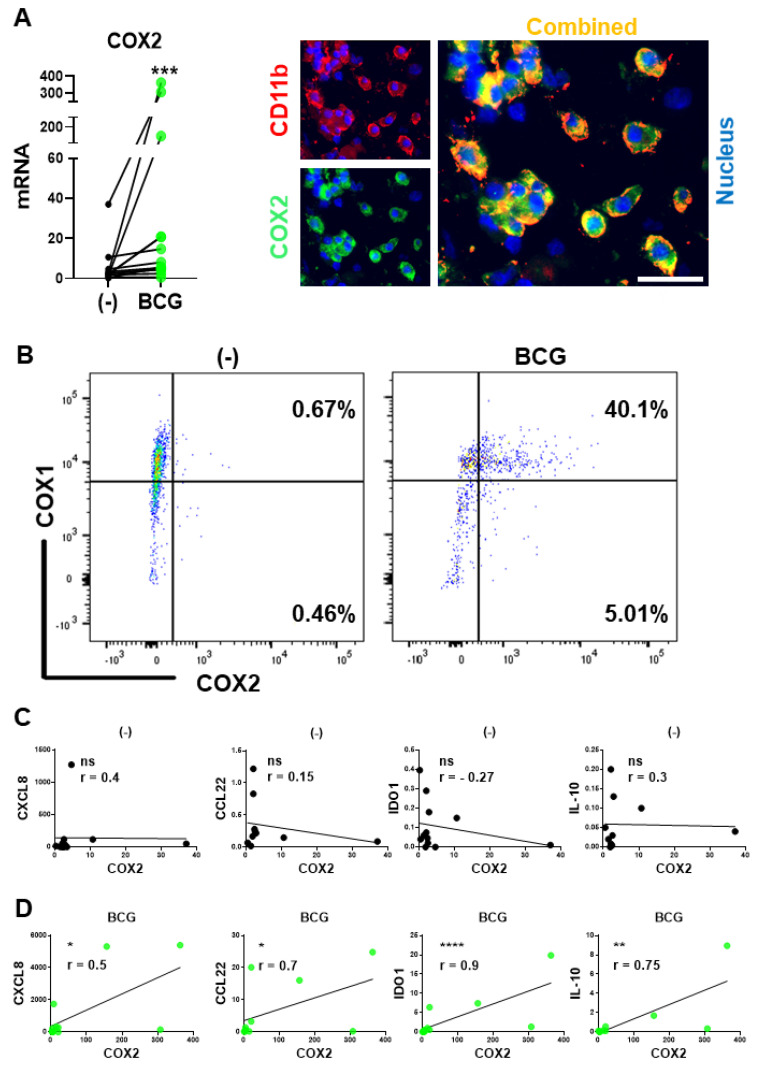
BCG induces COX2 expression: BCG-induced but not baseline COX2 levels correlate with intratumoral expression of immunosuppressive factors in BlCa microenvironments. (**A**)*, Left:* BCG enhances COX2 mRNA expression in ex vivo treated tumor tissues from BlCa patients (*n* = 13). Each pair of symbols and connecting line represents a single patient (mean mRNA expression in triplicate cultures per condition, relative to HPRT1). *Right:* CD11b^+^ myeloid cells are the major source of COX2 in BCG-treated BlCa TME. Tumor explants were treated for 24 h and evaluated by fluorescence microscopy. (**B**), Representative flow cytometry data from untreated and 24-h BCG-treated macrophage cultures from a single donor. (**C)**, Lack of correlations between COX2 and Treg/MDSC attractants (CCL22, CXCL8) or suppressive factors (IDO1, IL-10) in untreated BlCa tissues. (**D)**, Significant correlations between COX2 and CCL22, CXCL8, IDO1, and IL-10 expression in the BCG-treated BlCa explants. (**C**,**D**): Data are shown as ratios of the specific genes’ relative to HPRT1 expression determined in triplicate cultures of tumor tissues of 12 different BlCa patients (*n* = 12). Spearman r correlation is used for data evaluation. **** *p* < 0.0001; *** *p* < 0.001; ** *p* < 0.01; * *p* < 0.05.

Since we have recently demonstrated that the undesirable induction of multiple immunosuppressive factors by a commonly used immune adjuvant, poly-I:C, is mediated by COX2 and its product, PGE_2_ [34], we evaluated the levels of COX2 expression at the baseline and after BCG exposure. As shown in Figure 2, BCG treatment strongly enhanced the levels of COX2 expression in BlCa tissue explant cultures, being particularly pronounced in BlCa-infiltrating CD11b^+^ myeloid cells (Figure 2A), and in in vitro cultured human macrophages (Figure 2B).

Mouse in vivo models demonstrated that COX2 inhibition can counteract the growth of mouse BlCa tumors both in the absence and presence of BCG treatments [15,41,42,43], raising the question of whether the treatment-limiting effect of PGE_2_ results from aberrant baseline production of intratumoral PGE_2_ or from its induction by the BCG treatment. As shown in Figure 2C,D, the association between the COX2 and MDSCs/Treg attractants CXCL8 and CCL22, and suppressive factors IDO1 and IL-10 were observed exclusively after the BCG treatment of human BlCa tumor tissues, but not at baseline. These results indicate the key role of the undesirable component of BCG signaling in promoting local suppressive phenomena, indicating the importance of identification of the key pathways of BCG-driven PGE_2_ induction and signaling and potential for their targeting to enhance the therapeutic outcomes.

### 2.2. COX2 Induction by BCG Is Mediated by the NFκB Pathway

**Figure 3:**
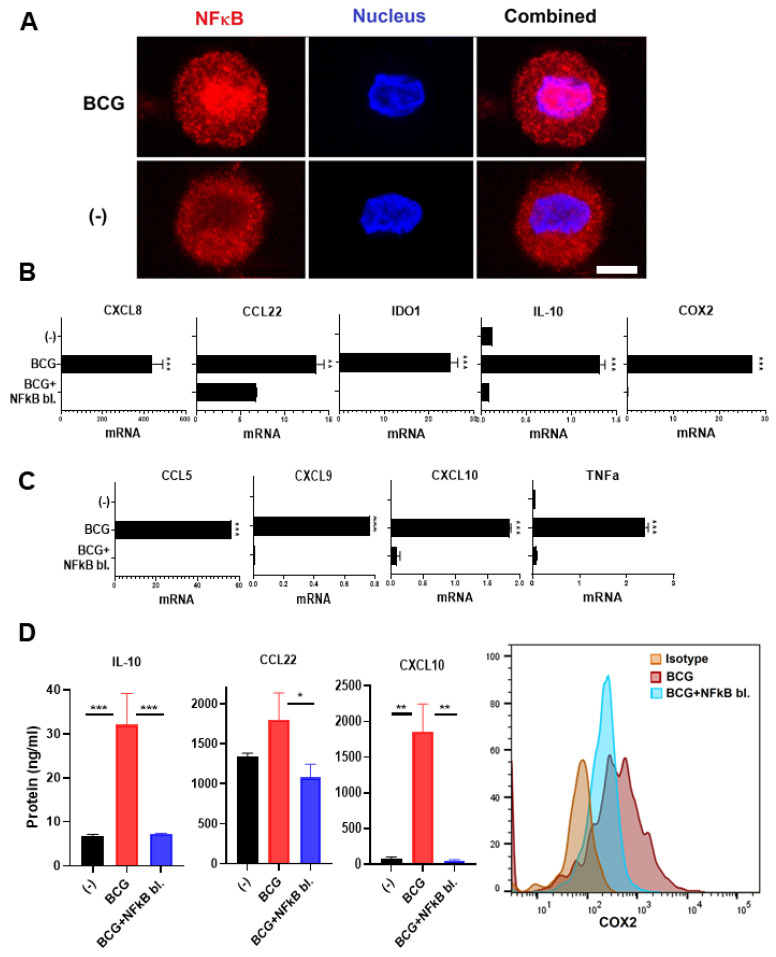
BCG-activated NFκB mediates the induction of TNFα and both immunostimulatory- and suppressive factors. (**A**), BCG induces NFκB nuclear translocation. Monocyte-derived macrophages were activated with BCG for 90 min and then NFκB was evaluated via confocal microscopy. NFκB blocker BAY11-7082 downregulates both (**B**), the Treg/MDSC attractants and suppressive factors CXCL8, CCL22, COX2, IDO1, and IL-10, as well as (**C**), CTL attractants CCL5, CXCL9, CXCL10, and TNFα in BCG-activated human macrophages. (**D**), BAY11-7082 eliminates the BCG-driven induction of IL-10, CCL22, CXCL10, and COX2 protein expression (*n* = 3). Data are mean ± SEM from at least triplicates per donor. Gene expressions are normalized relative to HPRT1. Wilcoxon rank test is used for statistical analysis. *** *p* < 0.001; ** *p* < 0.01; * *p* < 0.05.

Our current observations of the induction of TNFα by BCG (Figure 2) and our recent demonstration of the key role of the NFκB/TNFα axis in the induction of COX2 expression by poly-I:C, acting through the RIG-I/MDA5 pathway [34], raised a possibility that NFκB is also involved in the COX2 induction by BCG treatment. To address this possibility, we exposed human macrophages to BCG for 90 min, before staining for intracellular NFκB and analysis of the intracellular pattern of distribution by confocal microscopy. As shown in Figure 3A, BCG promoted nuclear translocation of NFκB.

To analyze the causative role of NFκB/TNFα and the induction of COX2 expression, we combined the BCG treatment with BAY11-7082, used in our previous studies to block NFκB phosphorylation and nuclear translocation in response to poly-I:C [34]. In accordance with the critical role of NFκB, BAY11-7082 completely blocked the BCG-driven induction of COX2, both at gene expression (Figure 3B) and protein (Figure 3D) levels, associated with the complete blockade of TNFα induction (Figure 3C).

The blocking of NFκB further abrogated the induction of the additional immunosuppressive factors IDO1 and IL-10 (Figure 2B), known to depend on COX2/PGE_2_ activity [34,44]. It also eliminated the induction of MDSCs/Tregs-attractants CXCL8 and CCL22 (Figure 3B–D). However, NFκB inhibition also blocked the production of CTL attractants (Figure 3C,D), demonstrating the key role of NFκB in the induction of both suppressive and effector cell-promoting factors by BCG and arguing against the combined use of BCG with NFκB blockade in the therapy of BCG.

### 2.3. Blockers of PGE_2_ Production or PGE_2_ Responsiveness Selectively Suppress the BCG-Driven Induction of MDSC/Treg Attractants and Immunosuppressive Factors, but Enhance the Secretion of CTL Attractants

The critical need for NFκB activity for intratumoral production of CTL attractants by BCG (Figure 3) and other factors [33,45] makes its inhibition undesirable in cancer immunotherapy. Since BCG induced COX2 expression (Figure 3B,D) and enhanced the expression of the key suppressive PGE_2_ receptor [46] EP4, but not EP2 (Figure S1), we tested if the magnitude and selectivity of action of BCG in inducing CTL attractants can be enhanced by targeting a downstream mediator of NFκB activity, PGE_2_ [34], by blocking its synthesis by indomethacin (COX1 and COX2 blocker) or celecoxib (selective COX2 blocker) or by blockade of PGE_2_ signaling, using EP4 receptor blocker, ARY-007.

**Figure 4:**
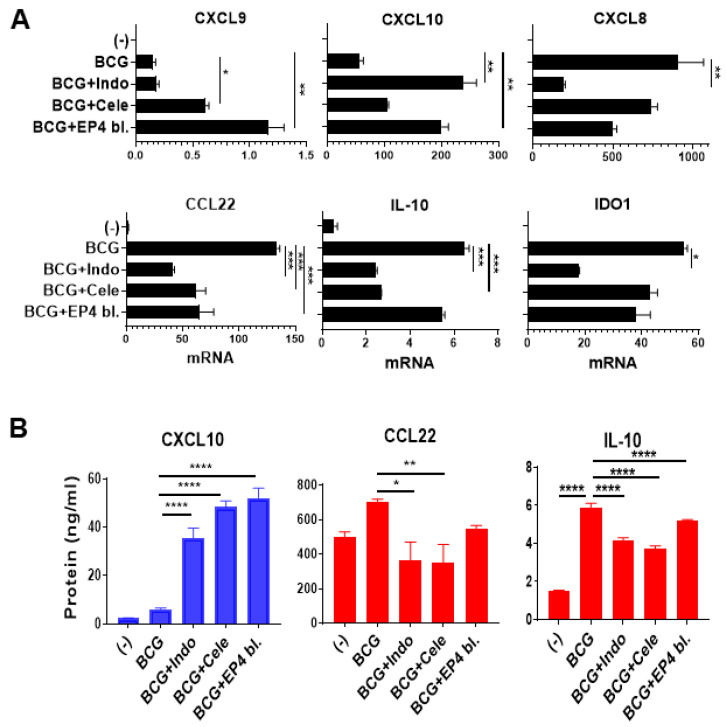
Combining BCG with the blockers of PGE_2_ synthesis or PGE_2_ signaling selectively enhances the desirable inflammatory factors in human macrophage cultures. (**A**), Combining BCG with indomethacin (Indo; COX1 and COX2 blocker), celecoxib (Cele; selective COX2 blocker) or ARY-007 (selective EP4 receptor blocker) enhances the induction of CTLs attractants; CXCL9 and CXCL10; but downregulates suppressive mediators; IDO1, IL-10, and MDSCs/Tregs-attractant; CXCL8 and CCL22. mRNA expression levels are normalized against HPRT1. (**B**), the combination therapy increases the secretion of CXCL10, but reduces CCL22 and IL-10 secretion by human macrophages. Data are shown as mean ± SEM. Wilcoxon rank test was used for statistical analysis. Results are representing the readings from human-grown macrophage culture from 3 different donors (*n* = 3). All cultures were performed in at least a triplicate per donor. **** *p* < 0.0001; *** *p* < 0.001; ** *p* < 0.01; * *p* < 0.05.

As shown in Figure 4A,B, all the above PGE_2_ antagonists selectively enhanced the CTLs-attracting chemokines, CXCL10 and CXCL9, but downregulated the MDSCs/Tregs-attracting chemokines, CXCL8 and CCL22. In the same uniform manner, the immunosuppressive factors, IDO1 and IL-10, were also suppressed by all the blockers of PGE_2_ synthesis and activity.

These data indicate that PGE_2_ antagonism may be superior to NFκB blockade in modulating the bladder cancer microenvironment, due to its unique ability to enhance both the overall potency and specificity of action of BCG.

### 2.4. PGE_2_ Antagonism Selectively Enhances CTLs Attraction to BCG-Treated Tumors, but Counteracts the Treg Attraction

**Figure 5:**
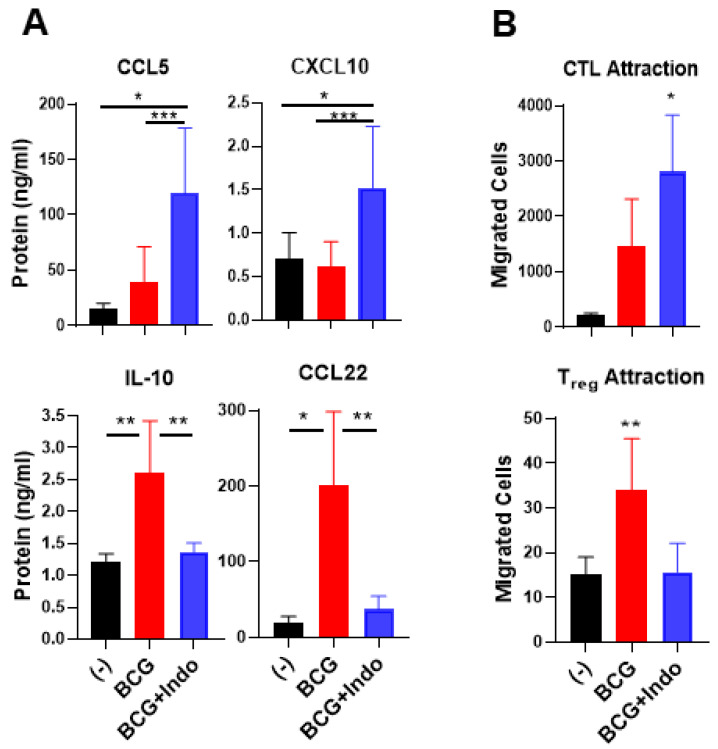
Combination of PGE_2_ antagonism with BCG selectively enhances the ability of BlCa tissues to attract cytotoxic T cells but reduces Treg attraction. (**A**), Ex vivo-cultured BlCa tumor tissues treated with BCG with indomethacin (Indo) showed significantly elevated secretion of CCL5 and CXCL10 but reduced secretion of IL-10 and CCL22 into tumor supernatants. Data are representative to tumor tissues from 11 different bladder cancer patients (*n* = 11). (**B**), Tumor explants treated with the combination therapy promoted preferential attraction of CTL rather than Tregs, compared to BCG monotherapy treatment. Ex vivo migration assay was performed by using dendritic cells-sensitized double positive CD8 ^+^/GZMB^+^ T cells as CTLs or CD4 ^+^/FoxP3^+^ T cells as Tregs, and supernatants from the differentially treated tumor tissues in the lower chamber from 3 independent bladder cancer patients (*n* = 3). The results are shown as mean ± SEM. Wilcoxon rank test is used for statistical analysis. All cultures were performed in at a least triplicate per patient. *** *p* < 0.001; ** *p* < 0.01; * *p* < 0.05.

To evaluate the functional relevance of these observations, we compared the impact of BCG versus BCG plus indomethacin on the ability of the differentially treated BlCa tumors to attract the above subsets of immune cells. Although BCG consistently upregulated the gene expression of several CTL-attracting chemokines (Figure 1A), BCG alone did not induce a consistent secretion of CCL5 or CXCL10; however, it significantly enhanced CCL22 and IL-10 secretion (Figure 5A). In sharp contrast, the combination of BCG with indomethacin resulted in selective elevation of CCL5 and CXCL10 secretion, compared to untreated or BCG-treated tumors and significantly downregulated the release of the Treg-attractant CCL22 and the suppressive cytokine IL-10 (Figure 5A).

In ex vivo chemotaxis studies, we tested the ability of supernatants from ex vivo cultured tumor tissues (from 3 different bladder cancer patients, *n* = 3) that were either untreated or treated with BCG alone or BCG plus indomethacin (as attractants) to attract effector type CD8^+^ T cells induced by SEB-loaded DC1s [33,47] and to attract Tregs present in blood-isolated CD4^+^ T cells. As shown in Figure 5B, supernatants from BlCa explants treated with BCG plus indomethacin consistently attracted higher numbers of cytotoxic T cells (identified as double-positive CD8 ^+^/GZMB^+^ T cells) than the supernatants from BCG-treated BlCa tissues. In contrast, BCG plus indomethacin-treated BlCa tumors attracted significantly lower numbers of regulatory T(reg) cells (identified as CD4 ^+^/FoxP3^+^ T cells) than the BCG-only-treated tumors, showing no increases in Treg attraction compared to the untreated tumors. Therefore, blocking PGE_2_ enhances both the overall magnitude of the BCG-induced CTL attraction and the selectivity of its effects in BlCa tissues.

## 3. Discussion

Our data demonstrate that, in addition to inducing CTL attractants and immunostimulatory factors in BlCa TME, BCG also induces massive release of Treg- and MDSC-attracting chemokines and suppressive factors. The observation of a strong correlation between COX2 and IL-10, IDO1, CXCL8, and CCL22 exclusively after BCG exposure (but not at baseline in unstimulated bladder tumor explants), and the elimination of the induction of these factors by blockers of PGE_2_ production or signaling helps to explain the role of PGE_2_ as a factor promoting the progression of BlCa and its resistance to BCG and other treatments observed in mouse models [15,41,42,43]. It also provides a direct rationale for PGE_2_ targeting to selectively enhance the influx of the desirable effector cells into human BlCa TME and the effectiveness of BCG-based immunotherapies in BlCa patients.

In accordance with the known involvement of TLR2, 4, 9, and Mincle which signal through MyD88, in the activation of the immune system by BCG [48,49,50,51], we observed that NFκB (which also induces TNFα) is an essential pathway for the induction of PGE_2_ and other tumor-promoting aspects of BCG-induced inflammation. In addition to its involvement in tumor-associated immune suppression, NFκB signaling in the TME also promotes metastasis and resistance to apoptosis [52,53,54,55,56]. Our current and previous studies show that the tumor-promoting aspects of NFκB/TNFα signaling uniformly depend on the induction of PGE_2_ production and subsequent signaling through EP4 [34,57].

**Figure 6:**
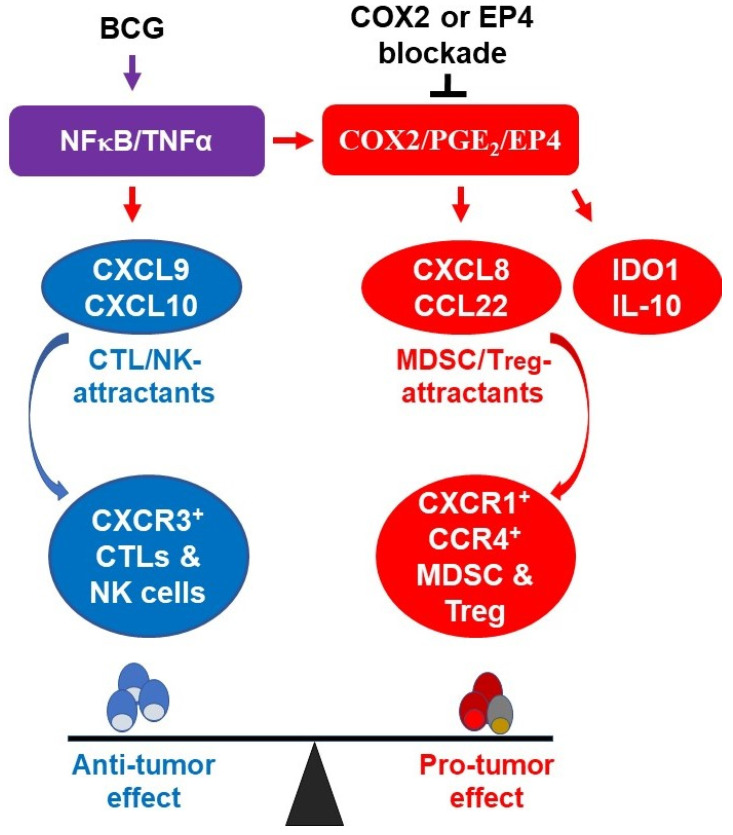
The molecular components of BCG-driven inflammation in bladder cancer microenvironment. BCG activates NFκB/TNFα signaling pathway which is responsible for the induction of CTL-promoting cytokines (CXCL9 and CXCL10), but also triggers the suppressive COX2/PGE_2_/EP4 pathway which includes MDSC/Treg-attracting chemokines (CXCL8 and CCL22) and additional immunosuppressive inflammatory mediators (IDO1 and IL-10). Combining BCG with indomethacin, celecoxib or EP4 blockade selectively enhances the CTL attraction but eliminates the PGE_2_ dependent suppressive factors, favoring antitumor immunity.

The TME is known to be an important venue of tumor escape from the immune system. Tumor-infiltrating Tregs play a tumor-promoting role through suppression of effector immunity [58,59,60]. The presence of Treg markers in the urine of BCG-treated BlCa patients has been shown to be associated with poor outcomes [61]. The infiltration of the bladder microenvironment by FoxP3^+^ T cells predicts BCG treatment failure and poor survival of BlCa patients [20]. Another population implicated in the current studies are the myeloid cells, which we have previously shown to require PGE_2_ for tumor infiltration and immunosuppressive activity [31,44]. Interestingly, mice receiving intradermal BCG have shown systemic increases in MDSC numbers [62], while the magnitude of MDSCs increases in urine after BCG administration reflects immune suppression and predicts poor recurrence-free survival [63]. We have previously shown that the enhanced production of CXCL12 in response to PGE_2_ exposure is associated with enhanced MDSCs accumulation in ovarian cancer microenvironments [31]. Therefore, our current results help explain the observations of enhanced MDSCs numbers in the urine of bladder cancer patients treated with BCG [63]. Additionally, a recent study indicated that CXCL8 and additional STAT3-dependent chemokines, CXCL1 and CXCL2) in activated bladder cancer microenvironment enhance the accumulation of neutrophils highlighting the pleiotropic character of inflammatory chemokines in shaping the bladder cancer microenvironment (Figure 6) [64].

In addition to the indication that PGE_2_ antagonism may increase the efficacy of BCG in early stages of the BlCa, our data suggest that the same combination may also enhance the effectiveness of PD-1/PD-L1 blockade in patients with muscle invasive or metastatic BlCa. PD-1/PD-L1 blockade has been approved for treatment of muscle invasive bladder cancer [65,66] and many other cancers, but its effectiveness is limited to the subset of patients with CTL infiltrated tumors [4,67]. Several clinical trials combining BCG with PD-1/PD-L1 blockade are ongoing, and it remains to be confirmed whether the effect of this treatment extends beyond the 25% of NMIBC patients with PD-L1-positive tumors [8].

The immunomodulatory effects of PGE_2_ are mediated by EP1, EP2, EP3 and EP4 receptors [46]. EP2 and EP4 mediate the key suppressive and tumor-promoting effects in many cancer types [68,69]. The co-overexpression of EP4 receptor, which we observed upon treatment with BCG (Figure S1), and COX2 have been shown to correlate with the poor overall survival of BlCa patients [70]. PGE_2_ drives the EP4-mediated elevation of COX2 and creates a positive feedback loop between COX2, PGE_2_ and EP4. This positive feedback loop is critical to driving intratumoral overproduction of not only PGE_2_ but also IDO1, IL-10 and ARG1 [44,71], which block the activation, expansion and killing functions of CTLs and NKs [44], and for enhanced of MDSCs and Tregs to the TME [71]. Therefore, our current data indicate that interference with PGE_2_ synthesis or signaling may allow to abrogate multiple aspects of the tumor-promoting activities in the BlCa TME, by targeting a single factor. While the number of patient samples in the current study did not allow us to determine any correlation between tumor stage and COX2 level, the data form the currently studied samples indicate a general advantage of the responses of bladder cancer microenvironments to combinatorial therapies involving BCG and PGE_2_ antagonists. This implication will be tested in our upcoming studies.

## 4. Materials and Methods

### 4.1. Study Design and Patients

Post-surgical bladder tumor tissues were obtained from 17 patients (*n* = 17) during the period of 2018–2020, with histologically confirmed BlCa; under Roswell Park Comprehensive Cancer Center Institutional Review Board approved protocol 098318. Written informed consent was obtained from all patients before collecting tissues. All studies were performed according to the World Medical Association Declaration of Helsinki. The patients’ clinical characteristics are given in Table 1.

### 4.2. Ex Vivo Human Bladder Tissue Cultures

The bladder tumor tissues were cut into 2 mm^3^ cubes using a sharp 4 mm sterile scalpel and cultured in AIM-V media (#12055091, Thermo Fisher Scientific, Cambridge, MA, USA) (around 5–7 cubes per well, 48 well plates) for 24 to 48 h under different conditions as described previously [7,33,34]. Each condition/treatment was set up at least in triplicates. Tissues were harvested for mRNA analysis, while supernatants were harvested for chemokine release analyses by ELISA and chemotactic assay [7,33,34].

### 4.3. Generation of Human Macrophages

Peripheral blood monocytes (PBMC) were collected from whole blood of healthy donors (*n* = 5) using the Ficoll separation method as described previously [34]. Monocytes isolated as light fraction of PBMCs through Percoll density gradient centrifugation were seeded in IMDM + 10% FBS + GM-CSF (1000 U/mL) at a density of 0.5 × 10^6^ cells in a 24 well plate for 6 days. On day 6, macrophages were treated with BCG with or without other blockers. After 24 h, cells were harvested for mRNA, confocal microscopy, or flow cytometry analysis, while supernatants were harvested for ELISA analysis and chemotaxis assay. Treatments were used according to doses previously established by our lab: TICE^®^ BCG (2 × 10^6^ CFU, Schering Plough, Newton, NJ, USA); Indomethacin, COX1/2 blocker (250 ng/mL, Millipore-Sigma, Germany); Celecoxib, specific COX2 blocker (10 mmol/L, Bio-vision, Milpitas, CA, USA); ARY-007, EP4 receptor blocker (250 ng/mL, KYN Therapeutics, Boston, MA, USA); BAY11-7082, NFκB inhibitor (10 µmol/L, Millipore-Sigma, Darmstadt, Germany) [7,34].

### 4.4. Generation of Activated CTLs

Pure CD8^+^ T cells were isolated from healthy donor PBMC by using the EasySep naïve CD8^+^ T cell enrichment kit (#19158, StemCell Technologies, Vancouver, BC, Canada). Monocytes-grown dendritic cells were loaded with staphylococcal enterotoxin B (SEB, 1 ng/mL). The previously isolated CD8^+^ T cells were co-cultured with SEB-loaded dendritic cells for six days. On day 7, pure T cells were harvested and adjusted to 10^6^ cells/mL for running the chemotaxis assay [33,47].

### 4.5. Chemotaxis of CTLs and Tregs

Twenty-four trans-well plates with polycarbonate filters (pore-size of 5 µm, #CLS3421, Millipore-Sigma, Darmstadt, Germany) were used for the immune cell chemotactic assay. The lower chamber was loaded with 400 µL of supernatants from differentially treated bladder tumor explants. 2 × 10^5^ CD8^+^ effector T cells suspended in 200 µL AIM-V were added to the upper chamber and allowed to migrate to the lower chamber for 90 min after which cells were harvested from the lower chamber, double-stained for CD8 and GZMB and analyzed by flow cytometry [33]. For the analysis of Treg migration, EasySep CD4^+^ T cell enrichment kit from StemCell Technologies (#17952, StemCell Technologies, Vancouver, BC, Canada) was used for the negative selection of Tregs from fresh PBMCs. 4 × 10^5^ CD4^+^ T cells were suspended in 200 µL AIM-V, added to the upper chamber and after 90 min the migrated cells were harvested from the lower chamber and stained for CD4 and FoxP3 and analyzed by flow cytometry [33,34].

### 4.6. Quantitative PCR Analysis of Gene Expression

Treated bladder tumor tissues and macrophages were collected and lysed by RLT buffer (#79216, RNeasy kit, Qiagen, Valencia, CA, USA) and homogenized to release their RNA contents. Afterward, supernatants were collected, and RNA was extracted using the RNeasy kit (#74106, Qiagen, Valencia, CA, USA). This was followed by cDNA synthesis using the Quanta biosciences synthesis kit (#101414-100). 25 ng of synthesized cDNA was measured and used for TaqMan mRNA analysis. The primers for chemokine mRNA analysis are CCL5 (#Hs00982282_m1), CXCL9 (#Hs00171065_m1), CXCL10 (#Hs00171042_m1), CXCL8 (#Hs00174103_m1), CXCL12 (#Hs03676656_mH) and CCL22 (#Hs01574247_m1). The analyzed mRNAs for PGE_2_ pathway are COX2 (#Hs00153133_m1) and EP4 (#Hs00168761_m1). And finally, the inflammatory mediators’ primers are IDO1 (#Hs00984148_m1), IL-10 (#Hs00961622_m1), NOS2 (#Hs01075529_m1) and TNFα (#Hs00174128_m1). HPRT1 primer (#Hs02800695_m1) was used as a housekeeping gene [7,34,72]. All TaqMan primers were obtained from Thermo Fisher Scientific Life Technologies, Cambridge, MA, USA.

### 4.7. ELISA Analysis of Secreted Chemokines

Chemokines released from treated macrophages or bladder tissue explants were identified by specific ELISAs for the key CTLs attractants (CCL5, #SRN00B and CXCL10, #SIP100), Tregs attractant (CCL22, #DMD00) and inflammatory mediator (IL-10, #DY217B). Specific antibodies were purchased from R&D systems Inc., Minneapolis, MN. Plates from Corning Inc. (Corning, NY, USA) were coated with 1µg/mL antibody overnight at room temperature. The next day the plates were washed and blocked by 4% BSA in PBS for 1 h. Then 10 µL/well of supernatants were incubated for another 2 h and then washed. Then, 0.5 µg/mL of biotinylated secondary antibody was added, and the plates were washed after 1 h of incubation. This was followed by the one-hour addition of Streptavidin-HRP conjugate (Thermo Fisher Pierce Biotechnology Inc., Rockford, IL, USA) for detection and then washed. After wash, TMB (3,3′,5,5′-Tetramethylbenzidine) substrate (Thermo Fisher Pierce Biotechnology Inc.) was added for thirty minutes to detect the formed complex. After 10–20 min, the reaction was stopped using 2% sulfuric acid and plates were analyzed at 450 nm by using a Multilabel Microplate Reader (Perkin Elmer, Waltham, MA, USA).

### 4.8. Flow Cytometry

Monocytes-grown macrophages were harvested after incubation for 24 h with treatments and intracellularly stained for anti-COX1-FITC/COX2-PE (undiluted, #334090, BD Biosciences, San Jose, CA, USA and surface stained for anti-EP4-PE (1:25, #10479, Cayman, Ann Arbor, MI, USA). After the chemotaxis assay, Tregs were stained for surface anti-CD4-PE (1:50, #IM0448U, Beckman Coulter, Brea, CA, USA) and intracellular anti-FoxP3-BV421 (1:50, #320214, Biolegend, San Diego, CA, USA), and CTLs were stained for surface anti-CD8-PE (1:25, #555369, BD Biosciences, San Jose, CA, USA) and intracellular anti-GZMB-FITC (1:25, #560211, BD Biosciences, San Jose, CA, USA). Stained cells were analyzed by LSRII A flow cytometry (BD Biosciences, San Jose, CA, USA) at the Roswell Park Flow and Image Cytometry Shared Resource (FICSR).

### 4.9. Immunofluorescence Imaging

For fluorescence microscopy imaging, tumor tissues were activated by BCG for 24 h and stained with anti-COX2-AF647 (1;50, #ab225273, Abcam, Cambridge, MA, USA) and anti-CD11b-PE (1:200, #553311, BD Bioscience, San Jose, CA, USA). Staining was performed according to our previous protocol [33]. Briefly, ex vivo cultured BlCa explants were embedded in optimum cutting temperature (OCT) medium, followed by freezing in methyl butane. Frozen tumor tissue was cut into 8-micron sections, fixed in acetone for 10 min, airdried, and stained with antibodies for 1–2 h. Imaging was performed using an ECHO, REVOLVE4 fluorescence microscope. For confocal microscopy imaging, macrophages were activated by BCG for 90 min and then stained for anti-Phospho-NFκB p65-AF488 (1:50, #4886, Cell Signaling, Danvers, MA, USA) according to the manufacturer protocol. Finally, imaging was performed using a Leica TCS SP8 Laser Scanning Spectral Confocal Microscope at the Roswell Park FICSR.

### 4.10. Statistical Analysis

All statistical analyses were conducted using GraphPad Prism 8.0 software. Non-parametric Wilcoxon matched paired signed rank was used to determine statistical significance between multiple treatment conditions of the same tumor or culture sample (**** *p* < 0.0001; *** *p* < 0.001; ** *p* < 0.01; * *p* < 0.05; not statistically significant (ns) *p* > 0.05). All data points were performed at least in triplicates. The number of patients or monocyte donors involved in the individual experiments are described in the individual figure legends. Spearman rank correlation (rho) was used for the analysis of the specified correlations between tumor-expressed genes.

## 5. Conclusions

Our data demonstrate that PGE_2_/EP4 axis limits intratumoral CTL attraction and promotes Tregs attraction and immune suppression in BCG-exposed human BlCa TME, indicating the feasibility of reprogramming BlCa for selectively enhanced CTL recruitment and reduced immune suppression.

## Figures and Tables

**Table 1:** Bladder cancer patients’ clinical data.

Clinicopathological Data	Patients, *n* = 17	
**Age (y), range: 54–90 y**	***n***	**%**
<65	6	35
≥65	11	65
**Sex**		
Male	10	59
Female	7	41
**Stage**		
0	3	18
I	0	0
II	7	41
III	7	41
IV	0	0
**Prior treatment**		
None	6	35
BCG	4	24
Chemotherapy	7	41

## Data Availability

This work involves commercially available reagents and materials, apart from ARY-007 obtained under an MTA from IKENA and the tumor samples obtained under an IRB approved protocol.

## Supplementary Materials

The following are available online at https://www.mdpi.com/2072-6694/13/6/1323/s1, Figure S1: BCG treatment elevates EP4 expression.
