# Supplementary material for: NFκB-Activated COX2/PGE2/EP4 Axis Controls the Magnitude and Selectivity of BCG-Induced Inflammation in Human Bladder Cancer Tissues
Source: Cancers (Basel). 2021 Mar 16;13(6):1323. doi: 10.3390/cancers13061323 (PMC7998891; doi:10.3390/cancers13061323)
Supplement: Supplementary file 1 [file cancers-13-01323-s001.pdf]

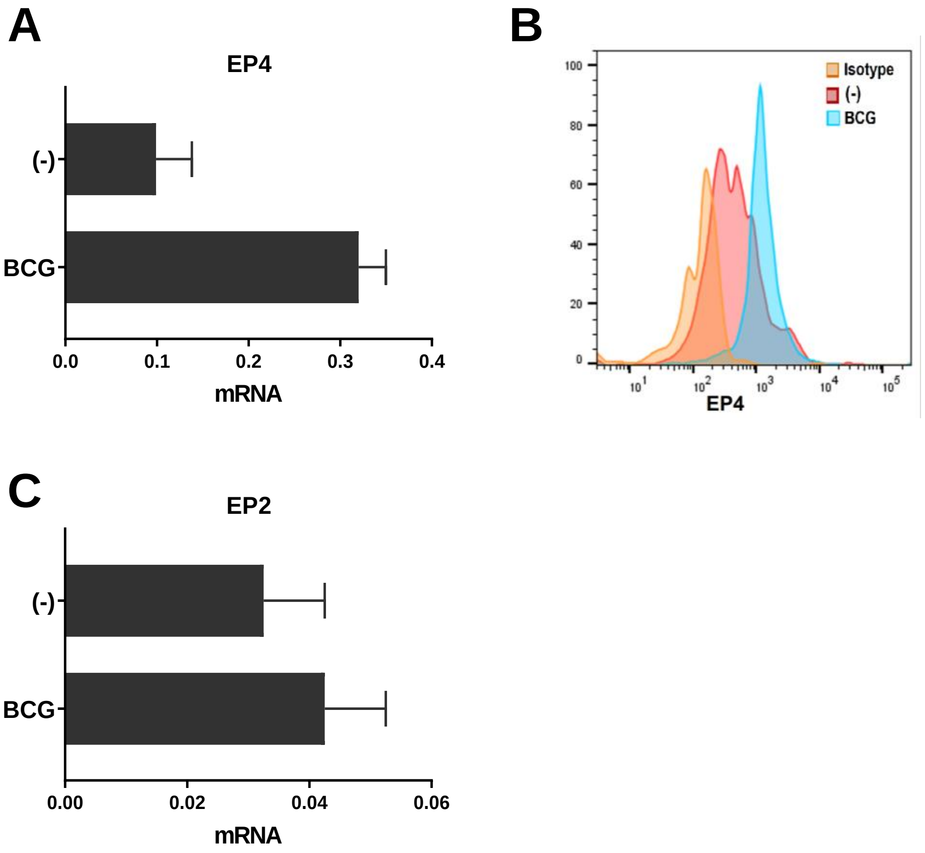

Supplemental Figure 1. BCG treatment elevates EP4 expression. A, BCG enhanced EP4 receptor gene expression in tumor tissue explants. **B,** EP4 receptor protein surface expression by cultured human macrophages measured by flow cytometry. Data from a representative donor. **C,** No changes in EP2 receptor gene expression in tumor tissue explants after BCG administration. Tumor tissue explants are from 4 different independent patients (*n=4*), The results are shown as mean ± SEM.
